# Cultural Variability in the Attribute Framing Effect

**DOI:** 10.3389/fpsyg.2021.754265

**Published:** 2021-12-20

**Authors:** Jeong Eun Cheon, Yeseul Nam, Kaylyn J. Kim, Hae In Lee, Haeyoung Gideon Park, Young-Hoon Kim

**Affiliations:** ^1^Social and Cultural Psychology Lab, Department of Psychology, Yonsei University, Seoul, South Korea; ^2^Social Cognition and Intergroup Perception Lab, Department of Psychology, The University of Utah, Salt Lake City, UT, United States

**Keywords:** framing effect, attribute framing effect, culture, regulatory focus, decision making

## Abstract

An intriguing phenomenon that arises from decision making is that the decision maker’s choice is often influenced by whether the option is presented in a positive or negative frame, even though the options are, *de facto*, identical to one another. Yet, the impact of such differential framing of equivalent information, referred to as the attribute framing effect, may not be the same for every culture; rather, some cultures may be more readily influenced by the differentially valenced frames than others (i.e., showing a greater difference in evaluation in a positive vs. negative frame). The present study investigates to what extent and why cultures may differ in their sensitivity to the attribute framing effect. Participants were recruited from South Korea and the United States, cultures characterized by their focus on prevention and promotion, respectively, to test for the cultural variability in the attribute framing effect. The results revealed that Korean participants were markedly more influenced by the valence of the frame than North American participants. Regulatory focus explained why Koreas showed a greater sensitivity toward the attribute framing effect than North Americans. Specifically, a greater prevention (vs. promotion) orientation of Korean participants led them to show a greater evaluation gap in the positive and negative frames. Implications for cultural significance on the attribute framing effect are discussed.

## Introduction

Is the glass half empty or half full? Although the two are logically identical states, how the glass is presented creates different impressions in people. The way the information is framed has important implications in shaping people’s evaluations and decisions, with even a slight change in information presentation embodying differing evaluative power (e.g., Levin, 1987; McKenzie and Nelson, 2003). Generally, a positively framed object or event (“glass half full”) gains a more favorable evaluation than if the same object or event was to be framed negatively (“glass half empty”). Such a tendency to attribute an aspect of information that looms larger in presentation, referred to as the attribute framing effect, has been theorized to take place because the way information is framed influences what becomes salient in people’s representation of the object or event (e.g., Levin and Gaeth, 1988; Igartua and Cheng, 2009); the positive aspect of information looms larger in memory when the information is presented positively, gaining a more favorable evaluation, whereas the negative aspect of the object or event is encoded in a negatively framed condition, leaving people to attribute unfavorable qualities to the object or event.

Although the literature on the framing effect is empirically well established over diverse domains including social, political, and economic decision making (e.g., McNeil et al., 1982; Chang et al., 2002), cultural differences in the effect of attribute framing have received little attention. Only recently, Nam et al. (2021) have pointed to the possible cultural variability in the derivation of the attribute framing effect. However, with no studies examining the differential impact and mechanism of the attribute framing effect on divergent cultures, it remains unknown why cultural differences may exist in the attribute framing effect and to what extent they may differ.

To promote a better understanding of the attribute framing effect, the present study aims to investigate to what extent and why cultures may be differentially influenced by the attribute framing effect. Specifically, the present study brings to bear the regulatory focus principle (Higgins, 1998, 2002) to investigate whether cultural differences in their orientation toward attaining desirable outcomes and avoiding undesirable outcomes specifically explain the power of the attribute framing effect. It is hypothesized that East Asians will display a greater sensitivity to the attribute framing effect as they have a greater prevention focus. North Americans, on the other hand, will be less influenced by the attribute framing bias as the cultural context North Americans are situated in leads them to develop a greater promotion focus.

### Regulatory Focus as an Explanatory Variable for Cultural Differences

Culture is the lens through which people view, evaluate, and decode information about the world (e.g., Hong et al., 2003; Nisbett and Miyamoto, 2005). Cultures come to emphasize different outlooks and behaviors in their socialization processes, thereby impacting on how people construe the world around them. The regulatory processes in which people avoid pain and approach pleasure are also likely to be heavily impacted by differences in cultural upbringing and atmosphere (Lee et al., 2000; Uskul et al., 2009). Indeed, previous studies have shown that cultures differ in their chronic regulatory focus (Kurman et al., 2015) or, more specifically, their tendency to avoid negative outcomes (a prevention focus) and approach positive outcomes (a promotion focus; Higgins, 1998). Specifically, in cultures where avoidance of losses and the fulfillment of obligations are emphasized, such as in East Asia (China, Japan, and South Korea; Lee et al., 2000; Kurman and Hui, 2011), people come to develop a chronic prevention focus (Higgins, 1998, 2002). In contrast, in cultures where the pursuit of gains and aspiration toward ideals are prioritized (Higgins, 1998, 2002), such as in the United States (Lee et al., 2000; Kurman and Hui, 2011), people develop a chronic promotion focus. Such cultural differences in regulatory focus are likely to explain why cultures may differ in their propensity to be influenced by differentially framed information. It is expected that in cultures where prevention focus is emphasized, people show a greater sensitivity to the attribute framing effect as it is prevention focus that is associated with a concrete processing style that renders them to be more sensitive to differentially contextualized information.

In detail, prevention-focused people may be more readily swayed by differentially contextualized cues because their motivation to avoid negative consequences leads them to adopt a concrete, localized, and specific perspective in navigating their environment (Förster and Higgins, 2005; Semin et al., 2005; Lee et al., 2010). Keen to screen possible threats or negativity in their environment (Higgins, 1998; Cunningham et al., 2005), prevention-focused people have shown to adopt a localized, lower-level perspective as opposed to a decontextualized, abstract mode of thinking (Förster and Higgins, 2005; Semin et al., 2005; Lee et al., 2010). Accordingly, in the decision-making context, prevention-focused people tend to center their attention on what looms largest or what seems the most certain in their thinking, rather than coming up with alternative explanations to ensure the best possible outcome (see Liberman et al., 2001; Molden et al., 2008). While conducive to scrutinizing threats, such strategies, evolved to minimize negative outcomes (vs. maximize positive outcomes), are likely to sensitize prevention-focused people to contextualized cues that are rendered larger in presentation. Indeed, attesting to such a tendency to be influenced by differentially valenced cues in their environment, prevention-focused people have been shown to grow readily unfavorable, becoming therefore vigilant in their decisions in the face of the negative stimuli (Higgins, 1998; Cunningham et al., 2005) while lowering their vigilance, even going further to change their evaluations and preferences in situations devoid of negativity (e.g., Roy and Naidoo, 2017).

On the other hand, promotion focus may be associated with a greater resistance to the attribute framing effect because, with the clear motivation to seek desirable outcomes (Higgins, 1998, 2002), promotion-focused people tend to perceive things more abstractly and globally (Förster and Higgins, 2005; Semin et al., 2005; Lee et al., 2010). That is, to acquire a desired end-state, promotion-focused people are required to incorporate various modes of thinking and adopt a global perspective (Förster and Higgins, 2005). Therefore, when making decisions, promotion-focused people tend to entertain much more alternative explanations in their thinking (Liberman et al., 2001) and adopt a higher-order, global perspective (Förster and Higgins, 2005; Semin et al., 2005) that allows them to move away from the contextual relations of the here and now that objects or events are often bound by (Trope and Liberman, 2010). In other words, promotion-focused people’s tendency to extract abstract logic in higher-order terms and generate many other alternative explanations in their thinking may insulate them from the influence of the attribute framing effect because the contextualized cue itself loses its significance when perceived further away.

On the basis of the tendency of prevention and promotion focus to attend to things differently, it is hypothesized that cultural differences in attribute framing effect stems, in part, from their differences in regulatory focus orientation. East Asians will display a greater sensitivity to the attribute framing effect because it is prevention-focused (vs. promotion-focused) individuals who are more severely impacted by differentially contextualized information. To test the hypothesis, the present study brings an accessible and practical platform of online reviews to calculate the impact of the framing effect separately for different cultures. By comparing and contrasting the impact of the framing effect, the present study aims to promote a practical understanding of the variability in its impact.

### Calculating the Variability Using Online Reviews

Before consumers purchase a product or service, they may often consider reading positive and negative reviews on an online platform. Generally, the more positive (vs. negative) reviews there are, the more likely consumers are to make a purchase (Park and Lee, 2008). Yet, at times, 60 positive (vs. 40 negative) reviews may be sufficient enough to bring about purchase behavior, whereas, at other times, 80 positive (vs. 20 negative) reviews are required to make people want to buy the product. One of the factors that works to lower or raise the bar in people’s purchase intention is the attribute framing effect (Putrevu, 2010; Jin et al., 2017). Because people become less strict in their evaluation when the item is positively framed (e.g., Levin and Gaeth, 1988), a smaller number of positive reviews should be sufficient to prompt purchase behaviors under the positive frame condition. On the other hand, as people tend to make harsher judgments on items that are negatively framed (e.g., Jin et al., 2017), more positive reviews are required to overcome the negatives and stimulate buying behavior. The increased degree to which positive reviews are required in the negative frame (vs. positive frame) condition reflects the power the framing effect has on people’s perceptions and behaviors.

However, hitherto unknown is the degree to which people with different cultural backgrounds and motivational outlooks change their evaluations as a consequence of differentially framing essentially the same information. As previous studies have often adopted a numerical anchor to manipulate the framing effect, e.g., 85% satisfied vs. 15% dissatisfied (Zhang and Buda, 1999) and 75% lean vs. 25% fat (Levin, 1987), the effect uniquely driven by the differential frames is not separable from the effect driven by the numerical anchor that also works to influence people’s decision making. That is, studies have shown that highly numerate individuals are, rather, insulated from the influence of the attribute framing effect (Kreiner and Gamliel, 2017) and, hence, conjoined usage of numerical and verbal anchors does not present researchers with the clearest picture on the power that differentially valenced frames have on different individuals.

In order to overcome the previous studies’ limitations and promote an in-depth understanding of the attribute framing effect, the present study implements an open-ended method to offer participants an opportunity to generate their own number of positive and negative reviews that needs to be encountered before participants ultimately decide to make a purchase. We refrain from using the numerical anchor that previous studies have used; instead, we use verbal anchors in asking people to freely estimate their threshold. A verbal anchor has been shown to provide people with a more intuitive understanding (Liu et al., 2020b) while being informative (Sanford and Moxey, 2003). Hence, it was in the interests of the present study to investigate people’s intuitive cognitive processes using an open-ended measure. Specifically, by refraining from using a numerical anchor, the present study allows people to generate their own threshold values using positive and negative verbal anchors (e.g., Liu et al., 2020a) by asking, what is the number of positive reviews it takes for customers to make the purchase (positive frame) and what is the number of negative reviews customers would allow to not be deterred from making the purchase (negative frame)? Both the smallest number of positive information necessary to make the purchase and the largest number of negative information that would deter people from making the purchase would indicate a tipping point in which the power of positive information can overcome the power of negative information in making a decision. By comparing people’s self-generated positive-to-negative ratio under differentially valenced frames, the present study aims to compare people’s tendency to be influenced by the attribute framing effect.

### The Present Study

The present research seeks to detect the differential impact of the attribute framing effect on different cultures. The regulatory focus principle is expected to explain why there may be cultural differences in the attribute framing effect. Specifically, it is hypothesized that a greater prevention focus explains why East Asians show a greater sensitivity to the attribute framing effect. On the other hand, a greater promotion focus is expected to explain why North Americans show a greater resistance to the attribute framing effect. Specifically, to clearly delineate cultural differences, the present study aims to test the hypothesis using people’s self-generated positive-to-negative ratio, a larger ratio signifying one’s higher sensitivity to the attribute framing effect.

In detail, the self-generated positive-to-negative ratio is calculated by asking participants to come up with the smallest number of “Recommended” reviews necessary (positive frame) or the largest number of “Not Recommended” reviews tolerable (negative frame) to make a purchase. Therefore, the studies were able to strip away any susceptibility to bias of numerical anchors and investigate the true influence of positive and negative frames. Using an open-ended measure, the study aimed to compare and contrast precisely the extent to which prevention focus (vs. promotion focus) shows greater sensitivity to the attribute framing effect, which should be evidenced by a greater positive-to-negative ratio in people’s responses.

**Figure 1:**
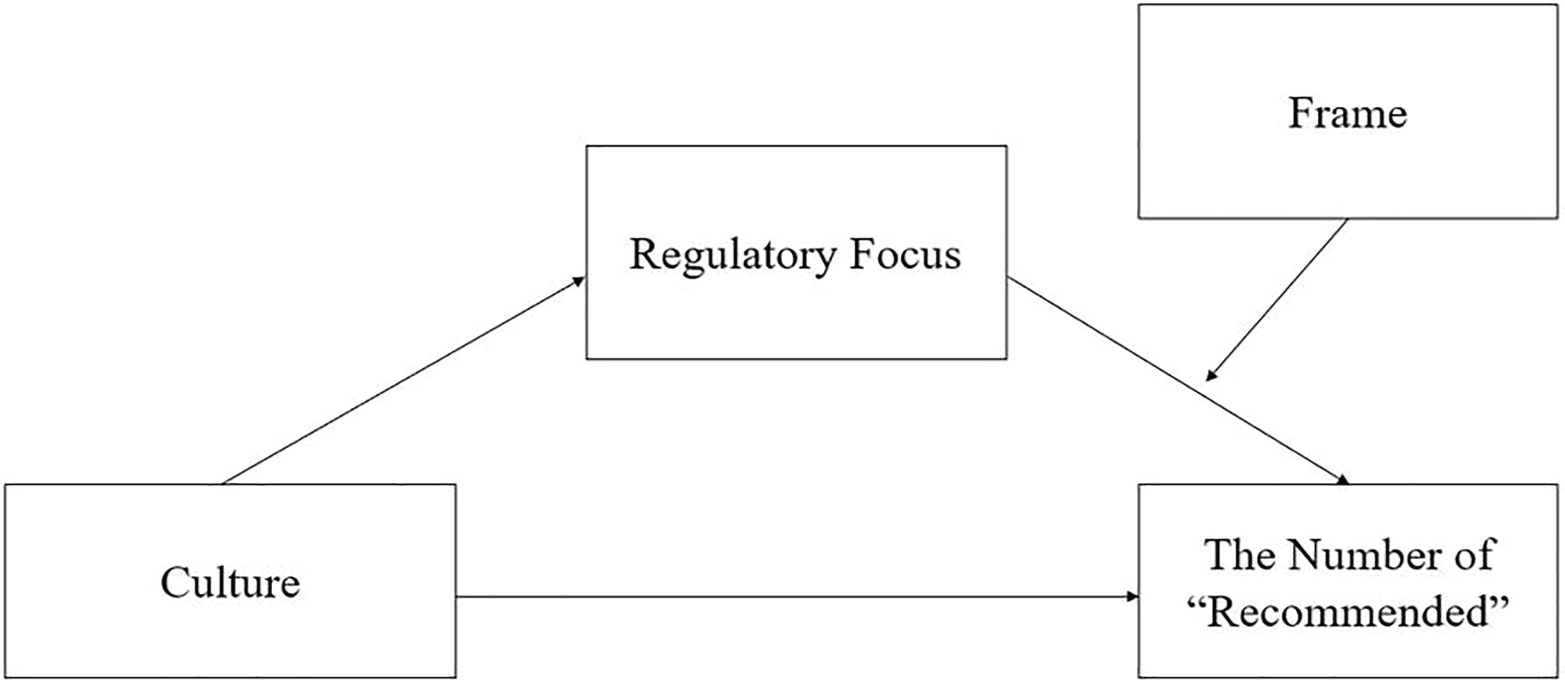
Moderated mediation model to be tested.

The specific model to be tested is presented in Figure 1. As shown in Figure 1, people from different cultures are expected to report different ratios of “Recommended” reviews necessary for purchase in positive and negative frames. Cultural differences in their evaluation gap in positive and negative frames are expected to be explained by their differences in regulatory focus orientation. To test for the model, Study 1 recruited participants from traditionally prevention- and promotion-focused cultures (East Asia vs. United States) to evaluate the impact of differentially framed information in shaping purchase intention, and Study 2 recruited participants from the same cultures for a replication using a different purchase domain.

## Study 1: Product Evaluation

### Method

#### Participants

Given the reported effect size of the attribute framing effect (*d* = 0.26; Piñon and Gambara, 2005), the present study recruited 600 American participants (277 men and 323 women) through Amazon MechanicalTurk (MTurk) and 947 Korean participants (455 men and 492 women) through DataSpring, a Korean online survey platform similar to MTurk. However, for those whose response duration failed to reach a minimum of 1 min, data were excluded for the analysis (United States: one man; Korea: eight men and one women). The mean age was 38.83 years (SD = 12.87) for the American participants and 29.71 years for the Korean participants (SD = 5.69). All participants completed the survey for a payment of approximately $1USD.

#### Manipulation

Participants were given an online scenario in which they were looking to purchase an everyday product, such as a calculator, an alarm clock, or a steam iron. The participants were told that the product had 100 binary reviews (either “Recommended” or “Not Recommended”) from other customers. Then, participants were asked to indicate the *minimum* number of “Recommended” reviews out of 100 required in order to purchase the product (*P*_recommend_; positive-framing condition) or the *maximum* number of “Not Recommended” reviews out of 100 that they would allow in order to purchase the product (*N*_not-recommend_; negative-framing condition). The dependent variable was the minimum number of “Recommended” reviews in the positive-framing condition and the maximum number of “Not Recommended” reviews in the negative-framing condition. Both the minimum number of “Recommended” reviews and the maximum number of “Not Recommended” reviews would serve to indicate the number of “Recommended” reviews required to make the purchase. Participants were randomly assigned to one of six conditions: 2 (frame: positive-framing vs. negative-framing) × 3 (product type: calculator, alarm clock, and steam iron).

#### Self-Regulatory Focus

To examine the moderating effect of self-regulatory focus (SRF) on the framing effect, four items were created to measure the extent to which participants were disposed toward either a promotion- or a prevention-focused approach. The following two items measured promotion focus: “It is more valuable for me to pursue success than to avoid failure” and “I prefer achieving desirable consequences to avoiding bad ones” (American: *α* = 0.85, Korean: *α* = 0.74). The following two items measured prevention focus: “It is more meaningful for me to avoid failures than to pursue successes” and “It is more important for me to avoid bad consequences than to achieve desirable ones” (American: *α* = 0.89, Korean: *α* = 0.64). Because we were interested in people’s predominant regulatory focus, we subtracted the total promotion-focused scores from the total prevention-focused scores. Higher scores represented individuals with a prevention-focused outlook and lower scores represented individuals with a promotion-focused outlook. This practice of calculating people’s predominant regulatory focus has been the standard approach in previous studies on regulatory focus (e.g., Uskul et al., 2009; Rodrigues et al., 2019).

### Results and Discussion

To compare the average number of “Recommended” and “Not Recommended” reviews that participants submitted, the number of “Not Recommended” reviews was subtracted from 100 (i.e., *N*_recommend_ = 100 − *N*_not-recommend_). Therefore, each participant’s response represented the number of “Recommended” reviews required to make the purchase, with higher numbers indicating a requirement for more favorable reviews.

#### Cultural Level Moderation Analysis

As the product type did not influence the results [culture × frame × product type being non-significant, *F*(8, 1,373) = 1.19, *p* = 0.30, *η_p_*^2^ < 0.01], the product type was collapsed in the analysis. First, we tested for the main effect of the frame manipulation (positive-framing condition = 1, negative-framing condition = 2) to replicate previous findings on the attribute framing effect. As expected, the main effect of the frame manipulation (positive-framing condition vs. negative-framing condition) was found to be significant, *F*(1, 1,381) = 103.54, *p* < 0.001, *η_p_*^2^ = 0.07. This finding indicated that participants from both cultures required more positive information when items were framed negatively (*M* = 77.73, SD = 20.05) than when framed positively (*M* = 63.02, SD = 27.46), which is in agreement with the previous studies on the attribute framing effect.

**Figure 2:**
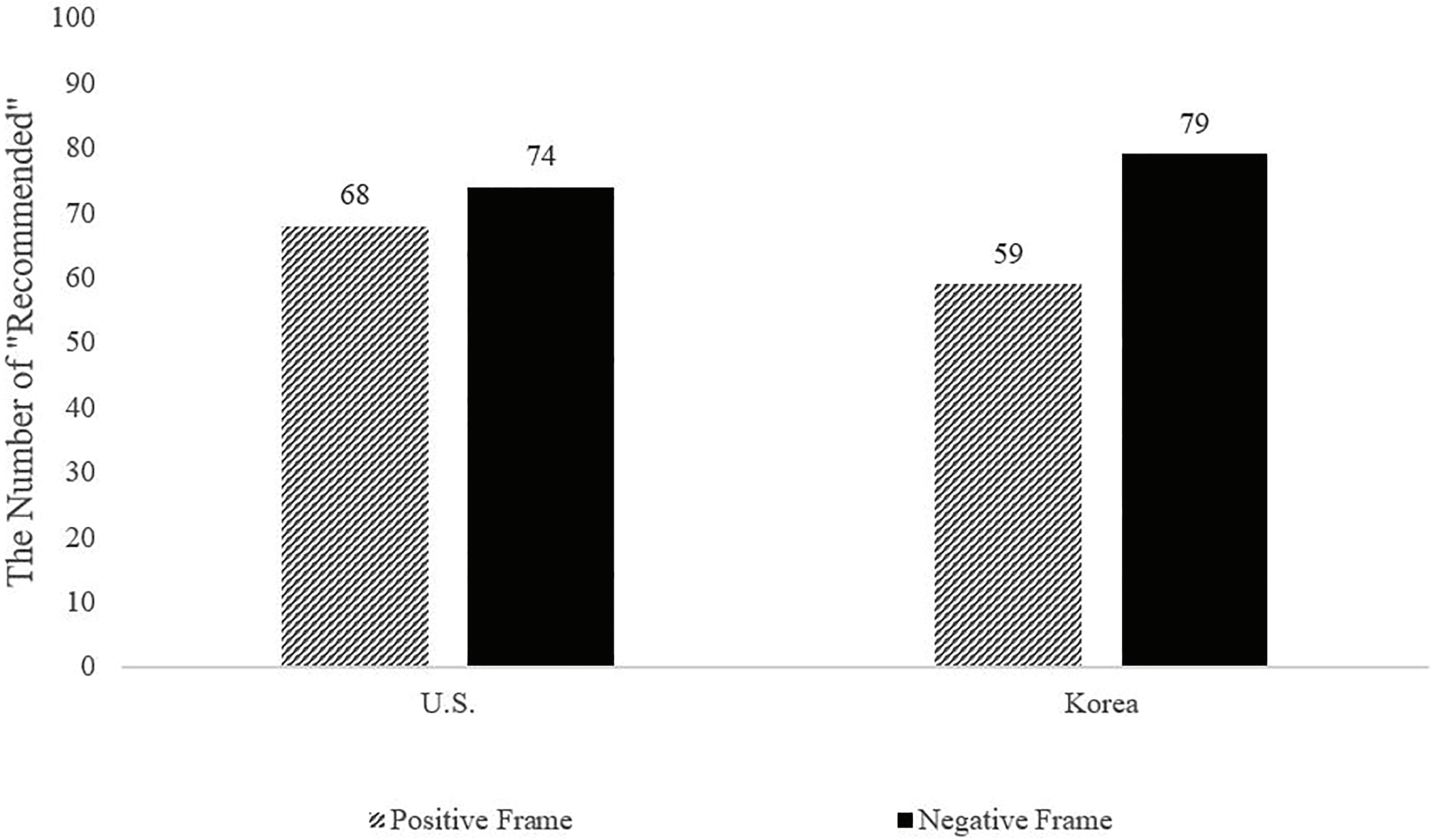
Infection effect of frame × country on the number of “Recommended” reviews needed for purchase (Study 1).

Before proceeding to investigate the moderated mediation model presented in Figure 1, we first tested for the moderation effect of culture (United States = 0, Korea = 1) in the relationship between valence of the frame and number of “Recommended” reviews needed for the purchase. Specifically, the study tested a 2 (culture: American vs. Korean) × 2 (frame: positive vs. negative) ANOVA on the number of “Recommended” reviews required. The interaction effect between culture and frame was significant, *F*(1, 1,381) = 27.07, *p* < 0.001, *η_p_*^2^ = 0.02 (see Figure 2), indicating that though there was a similar pattern of the framing effect for Americans and Koreans, the number of positive reviews needed to purchase the product in the negative-framing scenario rather than the positive-framing scenario was significantly greater for Koreans than for Americans.

Specifically, decomposing the significant interaction effect revealed that Korean participants reported that they need more “Recommended” reviews in the negative-framing (vs. positive-framing) condition, *t*(750) = −11.39, *p* < 0.001, *d* = 0.79, 95% confidence interval (CI) [−23.64, −16.69]. Seventy-nine “Recommended” reviews were needed in the negative frame condition (*N*_recommend_ = 79; SD = 20.53), whereas a minimum of 59 “Recommended” reviews were needed in the positive frame condition (*P*_recommend_ = 59; SD = 29.84). Comparing the number of “Recommended” reviews revealed that 34% more “Recommended” reviews were needed in the negative frame (vs. positive frame) condition (79/59 = 1.34).

American participants also reported a significantly greater number of positive reviews in the negative-framing (vs. positive-framing) condition, *t*(543) = −3.69, *p* < 0.001, *d* = 0.31, 95% CI [−10.00, −3.04]. However, the degree of difference between conditions was significantly lower as compared with that of Korean participants. Specifically, 74 “Recommended” reviews were needed in the negative-framing condition (*N*_recommend_ = 74; SD = 18.90) while 68 “Recommended” reviews were needed in the positive-framing condition (*P*_recommend_ = 68; SD = 22.63). That is, 9% more “Recommended” reviews were needed in the negative frame (vs. positive frame) condition (74/68 = 1.09).

In summary, Korean participants needed 34% more “Recommended” reviews, whereas American participants only needed 9% more “Recommended” reviews when they were presented with a negative (vs. positive) frame. Put differently, Koreans displayed 20-unit change in their answer (*N*_recommend_ = 79, *P*_recommend_ = 59), whereas Americans displayed only a 6-unit change in their answer (*N*_recommend_ = 74, *P*_recommend_ = 68). Consistent with the cultural hypothesis, a greater sensitivity to the framing effect was evidenced in Koreans whose cultural background is characterized by prevention-focus.

#### Moderated Mediation Analysis

To test the hypothesis that SRF would mediate the relationship between culture and the differential ratio of “Recommended” reviews required under the positive and negative frames, the moderated mediation model presented in Figure 1 was tested. The analysis was conducted using the PROCESS Model 14 by Hayes (2017). Five thousand bootstrapped resamples were used. Bootstrap confidence intervals were estimated for the indirect effect. Age and gender were controlled in the analysis.

The results revealed a significant index of moderated mediation (*B* = 1.76, boot SE = 0.68, 95% CI [0.51, 3.18]; see Table 1). As shown in Table 1, culture was significantly associated with a regulatory focus which, in turn, interacted with the valence of the frame (positive vs. negative) to influence the reported number of “Recommended” reviews necessary for purchase. Specifically, Koreans showed a greater prevention focus (*B* = 2.23, SE = 0.25, *p* < 0.001) which, in turn, was associated with a greater gap in their request for “Recommended” reviews under the positive and negative frame (Prevention: *P*_recommend_ = 60, *N*_recommend_ = 78; Promotion: *P*_recommend_ = 65, *N*_recommend_ = 76). Yet, interestingly, it was under the positive frame that the difference emerged (*B* = −1.34, boot SE = 0.56, 95% CI [−2.50, −0.27]). Under the negative frame, there was no significant difference between the promotion and prevention focus in their request for the number of “Recommended” reviews (*B* = 0.42, boot SE = 0.37, 95% CI [−0.27, 1.21]).

**Table 1:** Moderated mediation results of Study 1.

	Coefficient	SE	LLCI	ULCI
Outcome variable: regulatory focus (SRF)				
Culture	2.23	0.25	1.74	2.72
Age	−0.02	0.01	−0.05	0.00
Gender	−0.38	0.22	−0.81	0.05
Outcome variable: number of “Recommended”				
Culture	−0.25	1.51	−3.21	2.72
Regulatory focus (SRF)	−1.39	0.49	−2.35	−0.43
Valence	16.17	1.41	13.41	18.93
Regulatory focus (SRF) × Valence	0.79	0.30	0.20	1.38
Age	0.09	0.07	−0.05	0.23
Gender	−0.52	1.30	−3.07	2.02
Conditional indirect effects at different valence of frame	Bootstrapped Indirect effect	Boot SE	Boot LLCI	Boot ULCI
Positive frame	−1.34	0.57	−2.50	−0.27
Negative frame	0.42	0.37	−0.27	1.21

*SE, standard error; LLCI, lower limit confidence interval; and ULCI, upper limit confidence interval*.

The results revealed that a regulatory focus can explain why participants from different cultures display different sensitivity towards the attribute framing effect. That is, on the one hand, Korean participants displayed a greater prevention focus which, in turn, led them to show a greater evaluation gap in the negative and positive frame. On the other hand, American participants had a greater promotion focus and, subsequently, were less variable in their responses in the negative and positive frame.

## Study 2: Service Evaluation

Study 2 sought to replicate the findings in Study 1 by broadening the application scope. A hypothetical scenario in which participants were asked to imagine a situation where they were purchasing services online was presented. Specifically, the participants were asked to answer an open-ended question about the minimum number of positive reviews deemed necessary (positive frame) or the maximum number of negative reviews they would tolerate (negative frame) to hire someone for a service. Moreover, in order to increase the validity of results, Regulatory Focus Questionnaire (RFQ) of Higgins et al. (2001) was adopted for the study. This measure has been widely used in previous studies on regulatory focus. The usage of RFQ is expected to strengthen the results of Study 1.

### Method

#### Participants

Given that the degree of difference between cultures was found to be substantial in Study 1, a much smaller sample was expected to produce a reliable finding on the variability of the framing effect. Therefore, Study 2 recruited 200 American participants (122 men and 78 women) through Amazon MTurk and 236 participants (116 men and 120 women) through Embrain, a Korean platform similar to MTurk. As in Study 1, for those whose response duration failed to reach a minimum of 1 min, data were excluded from the analysis (Korea: five men and three women). The mean age of the American participants was 31.80 years (SD = 10.63), and for Korean participants, the mean age was 39.46 years (SD = 11.18). All participants completed the survey for a payment of approximately $1USD.

#### Procedure

The procedure was almost identical to the one administered in Study 1, with one change to the experiential stimuli. Instead of rating products, participants were given a scenario in which they would purchase a service online. Participants were randomly assigned to one of four conditions: 2 (frame: positive vs. negative) × 2 (service type: piano tutor, health trainer).

#### Manipulation

In the questionnaire, participants were given a scenario in which they were considering hiring someone as a music tutor or a personal trainer. Two different service roles ensured that the framing effect would be generalizable to different types. The participants were told that the tutor or trainer had received 100 binary reviews (either “Recommended” or “Not Recommended”) from other students or other trainees. In the negative-framing condition, they indicated the *maximum* number of “Not Recommended” reviews out of 100 that they would tolerate to hire someone for their services (*N*_not-recommend_). In the positive-framing condition, participants were asked to indicate the *minimum* number of “Recommended” reviews out of 100 they would require to hire someone for their services (*P*_recommend_). The dependent variable was the maximum number of “Not Recommended” reviews in the negative-framing condition and the minimum number of “Recommended” reviews in the positive-framing condition. As in Study 1, to compare the number of “Recommended” and “Not Recommended” reviews, the number of “Not Recommended” was subtracted from 100 so that each response represented the number of “Recommended” (*N*_recommend_ = 100 − *N*_not-recommend_).

#### Self-Regulatory Focus

Self-regulatory focus, the regulatory focus measure used in Study 1, as well as the RFQ of Higgins et al. (2001), was adopted to measure the individual level of regulatory focus. An additional measure of RFQ was used to ensure that the measure created in Study 1 (SRF) yielded a valid result.

Self-regulatory focus consisted of the same two items to measure promotion focus (American: *α* = 0.88, Korean: *α* = 0.78) and two items to measure prevention focus (American: *α* = 0.89, Korean: *α* = 0.69). RFQ consisted of six items measuring promotion focus (e.g., I feel like I have made progress toward being successful in my life; American: *α* = 0.74, Korean: *α* = 0.72) and five items measuring prevention focus (e.g., How often did you obey rules and regulation that were established by your parents?; American: *α* = 0.86, Korean: *α* = 0.73). The items were measured on a 7-point Likert scale. The promotion score was subtracted from the prevention score in order to match the results from Study 1.

### Results and Discussion

As in Study 1, the number of “Not Recommended” reviews was subtracted from 100 (i.e., *N*_recommend_ = 100 − *N*_not-recommend_) so that each participant’s response represented the number of “Recommended” reviews required to make the purchase.

#### Cultural Level Analysis

As service type did not interfere to change the result [2 (service type: piano tutor vs. health trainer) × 2 (culture: American vs. Korean) × 2 (frame: positive vs. negative) interaction being insignificant, *F*(4, 420) = 0.90, *p* = 0.47, *η_p_*^2^ < 0.01], two service roles were collapsed together for the analysis. As in Study 1, the main effect of frame manipulation (positive-framing condition = 1, negative-framing condition = 2) was significant, *F*(1, 424) = 666.58, *p* < 0.001, *η_p_*^2^ = 0.14. Participants in the negative-framing condition (*M* = 77.72, SD = 19.48) reported requiring a significantly greater number of “Recommended” reviews than those in the positive-framing condition (*M* = 59.38, SD = 29.23).

**Figure 3:**
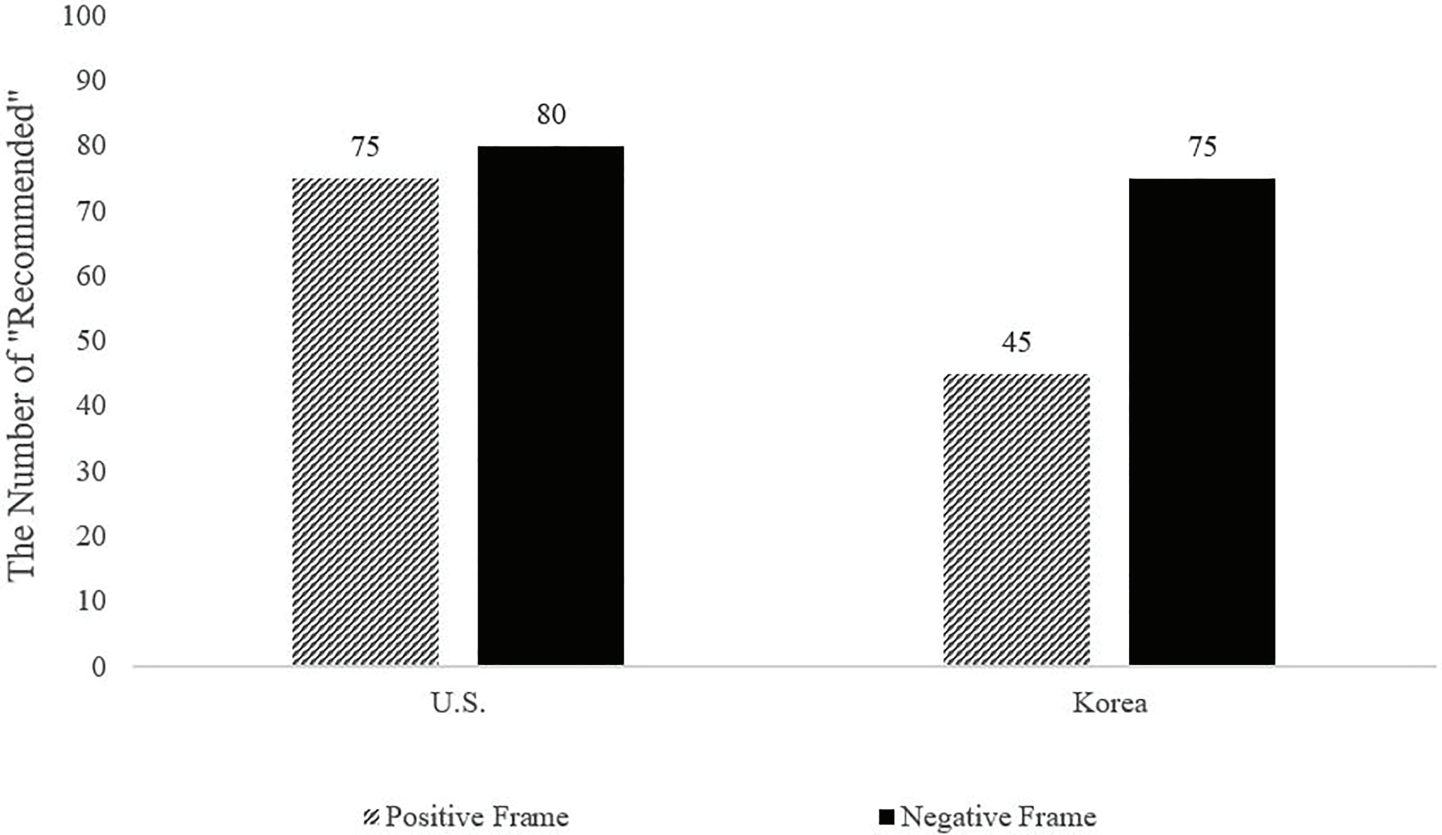
Infection effect of frame × country on the number of “Recommended” reviews needed for purchase (Study 2).

To test for the cultural variability in the attribute framing effect, we performed a 2 (culture: American vs. Korean) × 2 (frame: positive vs. negative) interaction. The interaction between culture (United States = 0, Korea = 1) and framing was found to be significant, *F*(1, 424) = 32.71, *p* < 0.001, *η_p_*^2^ = 0.07 (see Figure 3). As anticipated, the relative influence of the attribute framing effect was greater for Koreans than for Americans. Specifically, decomposing the significant interaction effect, Koreans reported that an average of 75 “Recommended” reviews were required in the negative-framing condition (*N*_recommend_ = 75; SD = 23.05), whereas 45 “Recommended” reviews were needed in the positive-framing condition (*P*_recommend_ = 45; SD = 31.76). That is, 67% more positive reviews were required in the negative-framing (vs. positive-framing) condition, *t*(206) = −8.22, *p* < 0.001, *d* = 1.09, 95% CI [−37.48, −22.99].

On the other hand, the difference was much smaller in American participants: Americans reported that they needed 80 “Recommended” reviews in the negative-framing condition (*N*_recommend_ = 80; SD = 13.81) and 75 “Recommended” reviews in the positive-framing condition (*P*_recommend_ = 75; SD = 14.69). Although significantly more positive reviews were required in the negative-framing condition, *t*(198) = −2.64, *p* < 0.01, *d* = 0.37, 95% CI [−9.29, −1.34], the resulting difference in the number of “Recommended” reviews was only 7% (80/75 = 1.07).

As in Study 1, the result revealed that a greater number of “Recommended” reviews were required in the negative-framing condition for both cultures, but the difference for the Korean participants was much greater than that for American participants (American: 107%, Korean: 167%). Although Koreans displayed a 30-unit change in their answer (*N*_recommend_ = 75, *P*_recommend_ = 45), Americans displayed only a 5-unit change in their answer (*N*_recommend_ = 80, *P*_recommend_ = 75).

#### Moderated Mediation Analysis

Next, we tested for the moderated mediation model presented in Figure 1. Two measures of regulatory focus (SRF and RFQ) were significantly correlated (*r* = 0.30, *p* < 0.001), yet a separate analysis was run for each regulatory focus measure for a clearer presentation of the results. As the interaction between frame manipulation and regulatory focus did not differ by the service type [SRF: *F*(1, 419) = 0.16, *p* = 0.69, Δ*R^2^* < 0.001; RFQ: *F*(1, 419) = 0.08, *p* = 0.78, Δ*R^2^* < 0.001], the service type was collapsed in the analysis.

First, we tested whether culture leads to SRF which, in turn, interacts with the valence of the frame to influence the number of the “Recommended” reviews necessary for purchase. In line with our expectations, the finding revealed a significant moderated mediation (*B* = 2.64, boot SE = 1.23, 95% CI [0.58, 5.39]; see Table 2). Specifically, Korean participants showed a greater predominant prevention focus (*B* = 1.23, SE = 0.23, *p* < 0.001) which, in turn, led to a greater evaluation gap in the positive and negative frame (Prevention: *P*_recommend_ = 53, *N*_recommend_ = 77; Promotion: *P*_recommend_ = 64, *N*_recommend_ = 78). As in Study 1, differences in the evaluation gap emerged in the positive frame condition (*B* = −2.94, boot SE = 1.03, 95% CI [−5.26, −1.20]) but not in the negative frame condition (*B* = −0.30, boot SE = 0.72, 95% CI [−1.67, 1.20]).

**Table 2:** Moderated mediation results of Study 2 (SRF as mediator).

	Coefficient	SE	LLCI	ULCI
Outcome variable: regulatory focus (SRF)				
Culture	1.23	0.23	0.77	1.70
Age	0.01	0.01	−0.01	0.03
Gender	0.18	0.23	−0.27	0.62
Outcome variable: number of “Recommended”				
Culture	−14.98	2.44	−19.78	−10.19
Regulatory focus (SRF)	−4.53	1.45	−7.37	−1.68
Valence	21.00	2.39	16.31	25.70
Regulatory focus (SRF) × Valence	2.14	0.95	0.28	4.01
Age	−0.14	0.10	−0.35	0.06
Gender	3.27	2.28	−1.21	7.75
Conditional indirect effects at different levels of valence	Bootstrapped Indirect effect	Boot SE	Boot LLCI	Boot ULCI
Positive frame	−2.94	1.03	−5.26	−1.20
Negative frame	−0.30	0.72	−1.67	1.20

*SE, standard error; LLCI, lower limit confidence interval; and ULCI, upper limit confidence interval*.

Next, the same analysis was conducted using the RFQ measure. The moderated mediation analysis was significant (*B* = 3.67, boot SE = 1.43, 95% CI [1.17, 6.75]; see Table 3). Korean participants showed a greater prevention focus while US participants showed a greater promotion focus (*B* = 0.85, SE = 0.13, *p* < 0.001). Subsequently, the regulatory focus orientation interacted with the valence of the frame to influence people’s responses regarding “Recommended” reviews necessary for purchase (*B* = 4.32, SE = 1.68, *p* = 0.01). In detail, the prevention focus was associated with a greater evaluation gap under the positive and negative frame (*P*_recommend_ = 54, *N*_recommend_ = 79) than the promotion focus (*P*_recommend_ = 63, *N*_recommend_ = 79).

**Table 3:** Moderated mediation results of Study 2 (RFQ as mediator).

	Coefficient	SE	LLCI	ULCI
Outcome variable: regulatory focus (RFQ)				
Culture	0.85	0.13	0.59	1.11
Age	−0.00	0.01	−0.01	0.01
Gender	0.16	0.13	−0.08	0.41
Outcome variable: number of “Recommended”				
Culture	−16.01	2.49	−20.91	−11.11
Regulatory focus (RFQ)	−7.60	2.58	−12.68	−2.52
Valence	17.58	2.27	13.11	22.05
Regulatory focus (RFQ) × Valence	4.32	1.68	1.03	7.62
Age	−0.15	0.10	−0.35	0.06
Gender	3.00	2.30	−1.52	7.52
Conditional indirect effects at different levels of valence	Bootstrapped Indirect effect	Boot SE	Boot LLCI	Boot ULCI
Positive frame	−2.78	1.14	−5.25	−0.80
Negative frame	0.89	0.81	−0.55	2.62

*SE, standard error; LLCI, lower limit confidence interval; and ULCI, upper limit confidence interval*.

In line with the result found in Study 1, Study 2 demonstrated that Koreans displayed a greater evaluation gap in the positive and negative frame, showing a greater sensitivity to the attribute framing effect. US participants, on the other hand, showed less difference in their response in the positive and negative frame. Predominant regulatory focus orientation explained this cultural difference. That is, Koreans showed a greater predominant prevention focus which, in turn, led them to make different responses in the positive and negative frame to a greater extent. US participants showed a greater predominant promotion focus which, in turn, led them to make relatively more similar responses in the positive and negative frame.

## General Discussion

The purpose of the two studies was to investigate the extent to which different cultures are influenced by the attribute framing effect and to further delineate its mechanism. It was hypothesized that those from Korea (vs. United States) would be more heavily impacted by differential valences of the frames on which equivalent information is presented due to their greater prevention focus (vs. promotion focus) orientation. Following the predictions, two studies confirmed that Koreans showed a greater sensitivity to the attribute framing effect as their greater prevention focus rendered them to be more sensitive to differentially contextualized information. Americans, on the other hand, were less affected by the attribute framing effect, showing a smaller evaluation gap in the positive and negative frames. It was a greater promotion focus of Americans that explained why they made relatively more similar responses in the positive and negative frames.

Specifically, when asked to quantify the number of favorable (vs. unfavorable) reviews necessary to make the purchase, Korean participants’ evaluation differed substantially more, depending on the valence of the frame the information was presented in. That is, Korean participants requested 20–30 more favorable reviews (out of 100) in the negative-framing condition than in the positive-framing condition. This is in stark contrast to American participants who requested only 5–6 more favorable reviews in the negative-framing (vs. positive-framing) condition. Such a difference in the sensitivity to the attribute framing effect was explained by cultural variability in their chronic regulatory focus. That is, Koreans showed a greater prevention focus which, in turn, affected the extent to which they were differentially impacted by positive and negative frames. A greater prevention focus was associated with a greater difference in people’s evaluation in the positive and negative frame. On the other hand, a greater promotion focus was associated with lower variability in people’s response in the positive and negative frame; it explained why American participants were, rather, shielded from the effect of the attribute framing.

However, unexpectedly, the differences in promotion- and prevention-focused individuals’ responses came from the positive frame. Specifically, while promotion- and prevention-focused people requested a similar number of “Recommended” reviews in the negative frame, it was prevention-focused people who requested much fewer “Recommended” reviews in the positive frame. This indicates that the prevention-focused is more prone to fall into the attribute framing bias because the *absence* of negative information allows them to become much more generous in their evaluations and responses. This is in line with finding of Nam et al. (2021) which indicated that the attribute framing effect stems from the absence of negative information for Koreans. Future studies may want to explore further the relationship between prevention focus and attentional bias in the absence (vs. presence) of negative information.

Moreover, another important point to note is that Americans generally requested more reviews than Korean participants. Although we were interested in the differential ratio of reviews necessary in the positive and negative frame, the absolute degree to which Americans requested more reviews merits further discussion. There are two plausible explanations for this. First, American participants’ requests for more reviews may merely reflect cultural differences in response styles. Previous studies have shown that those from the West tend to give more extreme answers than those from the East (Harzing et al., 2012). Thus, the mean score difference may not truly reflect their need for more information but, rather, their tendency to report higher numbers. In this sense, it is important to compare and contrast proportional differences between conditions rather than mean score difference *per se*, as was done in the present study. Second, the higher score may reflect the actual need of Americans to view more positive reviews or information in general. Because Americans are more promotion-focused than Koreans, they may come to demand more information to make the best possible choice. Indeed, their higher individualistic characteristic makes them to be responsible for their own actions (Waterman, 1981), which may lead them to require more information when making decisions.

Although presenting frames with differential valence have been shown to influence people’s perceptions and decisions (Ferguson and Gallagher, 2007; Sparks and Browning, 2011), in which cultures the framing effect is more powerful, and why it is more powerful, has not been fully understood. By delineating the degree to which people with different cultural backgrounds are impacted by positive and negative frames, the present study has promoted a better understanding of the variability of the framing effect. That is, the differential valence of the frame has been shown to have a much greater impact on Koreans than Americans. The variability in its impact is, as matter of fact, not negligible. For instance, in Study 2, Korean participants requested a six times greater number of positive reviews in the negative-framing (vs. positive-framing) condition. Such variability clearly speaks to the differential impact positive- and negative-framed messages should have on prevention-oriented (vs. promotion-oriented) cultures.

The significant difference in people’s sensitivity to the framing effect has important implications in a globalized world where standardized messages are readily being incorporated into divergent contexts. The present study alerts people that the use of standardized messages, irrespective of one’s regulatory orientation, may be venturesome. That is, while the choice of positive/negative words is expected to have a smaller impact in promotion-oriented culture, adopting the same message without paying special attention to the valence of the words and connotations in prevention-focused cultural contexts may drastically influence people’s decision in an unanticipated way. Therefore, careful adaptation of the messages is recommended.

However, at the same time, it is important to note that these findings must be considered within the context of the study’s limitation. For one, product purchase is not a one-dimensional experience; along with reviews, there are other factors, such as brands, price, and promotions (Gupta, 1988) that influence the customer’s decision that the present study has not taken into consideration. Although the present study aimed to clarify the unique effect of positive/negative reviews on different cultures and individuals, taking into consideration other factors that influence purchase intention will broaden the understanding of the issue. Second limitation is the study’s reliance on self-reports of purchase intentions, rather than observing actual purchase behavior. Although customers can predict their behaviors and future decisions to some extent, the intentions of their decisions do not necessarily equate to true purchase decisions, as it has shown to occasionally misalign (Morrison, 1979). In fact, people may actually not be able to accurately predict their behaviors based on introspection. Even so, we believe an insight into purchase intentions and the decision-making processes are a valuable contribution to the attribute framing literature. It is a worthy pursuit to understand how various cultures interpret new information in positive and negative frames. Third, we subtracted the promotion focus score from the prevention focus score to measure people’s predominant regulatory focus orientation. Although this practice of calculating predominant regulatory focus has been used previously in numerous studies (e.g., Uskul et al., 2009; Rodrigues et al., 2019), a more precise method of estimating one’s predominant regulatory focus should add to the study and future research on regulatory focus. Lastly, there are other factors that may interfere with the result of the study that we have not controlled for. For instance, educational level, income, and other psychological factors may intervene to influence the result. Future studies should measure such variables more comprehensively to ensure equality of comparison of participants from different cultures and to increase the internal validity of the result.

Despite these limitations, this investigation offers many strengths and a contribution to the existing attribute framing literature. Understanding the upper and lower thresholds for the accepted amount of positive and negative information is invaluable information for those who rely on messages for persuasion and influence. By knowing for whom, and to what extent, the framing effect has a greater impact, people can increase their confidence in the messages’ persuasiveness and effectiveness.

## Conclusion

Information framed positively begets positive evaluation, whereas the same information presented negatively begets negative evaluation. However, not all people are equivocally swayed by the valence of the frame in which the equivalent information is presented. To promote a better understanding of the variability in its impact, the present study investigated the differential impact of positive/negative frames in predicting purchase intention of people from different cultures. The mediating role of regulatory focus was examined to investigate whether cultural differences in their prevention and promotion orientation explain why they have different levels of sensitivity to the attribute framing effect. Two studies demonstrated that Koreans are more vulnerable to the influence of differential valences of the frame than Americans. That is, Koreans displayed a greater evaluation gap in their judgment depending on the valence (positive/negative) of the frame they were presented with. Further moderated mediation analysis revealed that prevention focus explained why Koreans display a greater susceptibility to the attribute framing. For Americans, the degrees to which valence of the frame influenced their judgment were substantially smaller. It was Americans’ higher promotion focus that partially shielded them from the influence of the attribute framing effect.

## Data Availability Statement

The raw data supporting the conclusions of this article will be made available by the authors, without undue reservation.

## Ethics Statement

The studies involving human participants were reviewed and approved by the Yonsei University Institutional Review Board. Written informed consent for participation was not required for this study in accordance with the national legislation and the institutional requirements.

## Author Contributions

All authors were involved in designing the study and procedures. YN collected the data. JC, YN, and KK analyzed the data and drafted the manuscript with input from HL and HP. YH-K supervised all of this work, and reviewed, edited, and finalized the manuscript. All authors contributed to the article and approved the submitted version.

## Funding

This research was supported by the Basic Science Research Program through the National Research Foundation of Korea (NRF) funded by the Ministry of Education (NRF-2018S1A3A2075114). This research was also supported by the Yonsei Signature Research Cluster Program of 2021-22-0005.

## Conflict of Interest

The authors declare that the research was conducted in the absence of any commercial or financial relationships that could be construed as a potential conflict of interest.

## Publisher’s Note

All claims expressed in this article are solely those of the authors and do not necessarily represent those of their affiliated organizations, or those of the publisher, the editors and the reviewers. Any product that may be evaluated in this article, or claim that may be made by its manufacturer, is not guaranteed or endorsed by the publisher.
